# Separating extracellular from intracellular fecal metabolites exposes cross-feeding architecture in the gut: exploring metabolite partitioning and ecological associations

**DOI:** 10.1080/19490976.2026.2719062

**Published:** 2026-08-24

**Authors:** Masaaki Hirayama, Fuka Takame, Tetsuya Maeda, Kenichi Kashihara, Mikako Ito, Kinji Ohno, Jun Ueyama

**Affiliations:** a Department of Occupational Therapy, Chubu University College of Life and Health Sciences, Kasugai, Japan; b Kirin Beverage Co., Ltd., Chubu Regional Headquarters, Nagoya, Japan; c Department of Internal Medicine, School of Medicine, Division of Neurology and Gerontology, Iwate Medical University, Iwate, Japan; d Okayama Neurology Clinic, Okayama, Japan; e Division of Neurogenetics, Center for Neurological Diseases and Cancer, Nagoya University Graduate School of Medicine, Nagoya, Japan; f Graduate School of Nutritional Sciences, Nagoya University of Arts and Sciences, Nisshin, Japan; g Department of Pathophysiological Laboratory Sciences, Nagoya University Graduate School of Medicine, Nagoya, Japan

**Keywords:** Vitamin, gut microbiota, biotin, *Alistipes*, cross-feeding

## Abstract

The gut microbiota forms a complex ecosystem through metabolic interdependence (cross-feeding). However, conventional fecal metabolomics typically quantifies only total metabolite pools, making it difficult to distinguish extracellular-enriched metabolite pools from predominantly cell-associated ones, and thus limiting reconstruction of in situ metabolic networks. Here, we quantified fecal metabolites in paired fractions from the same specimen: an undisrupted fraction representing the extracellular pool and a strongly bead-beaten fraction representing the total pool; the intracellular pool was defined as the difference between total and extracellular measurements. Stool samples from 63 healthy individuals were analyzed for short-chain fatty acids, polyamines, and water-soluble vitamins, and the results were integrated with shotgun metagenomic profiles of species composition and functional genes. Vitamins exhibited two distinct behaviors. Adenosylcobalamin (a vitamin B12 coenzyme) was detectable only after disruption, and together with thiamine (B1) and niacin (B3) was classified as an intracellular-retained type (Type 1). In contrast, biotin (B7) and pantothenate (B5) showed higher extracellular proportions (Type 2). Integrative analyses further indicated that Type 1 thiamine was negatively associated with *Blautia*, consistent with intensive microbial utilization, whereas Type 2 biotin was strongly positively associated with *Alistipes*, suggesting links to ecological niches shaped by luminal pH and fermentation modes (carbohydrate vs protein fermentation). Fraction-resolved quantification provides a practical operational framework to differentiate extracellular from cell-associated metabolite pools, helping reconcile metagenomic potential with metabolomic reality and enabling deeper inference of cross-feeding structure in the gut ecosystem.

## Introduction

The colonic lumen is a strictly anaerobic environment where the energy acquisition of the gut microbiota primarily depends on fermentation. Because oxidative phosphorylation based on oxygen respiration is unavailable, the available free energy is relatively limited. Consequently, gut bacteria form ecological niches through the optimization of substrate utilization, metabolite excretion, and symbiotic relationships.^1^ ^,^ ^2^ The primary carbon sources are indigestible carbohydrates (dietary fiber) and mucin-derived glycans supplied from the intestinal mucosa,^3^ the degradation of which dictates the material flux and the formation of the metabolite pool within the gut microbiota.

Intermediate metabolites and end products generated during fermentation are not only released into the environment through active secretion but can also be supplied to the intestinal lumen through the leakage of intracellular components following bacterial death or lysis.^4^ As a result, diverse low-molecular-weight compounds exist in the feces and can function as shared resources for the microbial community. In such an environment, evolutionary strategies favoring the uptake of externally available molecules are often selected over the maintenance of all biosynthetic pathways within a single cell. Consequently, lineages that partially lack biosynthetic capabilities for coenzymes (vitamins) or amino acids and rely on their surroundings (auxotrophy) are widespread.^5^

This external dependency drives cross-feeding (the mutual utilization of metabolites) within the gut microbiota, forming a community-wide metabolic network. Short-chain fatty acids (SCFAs; acetate, propionate, and butyrate) represent a typical group of such metabolites. Their production is supported by multi-step processes in which intermediate metabolites, such as lactate produced during glycolysis, are utilized by other species and converted into end products.^6^ Therefore, fecal SCFA levels should be understood not only as a reflection of production capacity but also as a community phenotype determined by the balance between host absorption and inter-microbial conversion.^7^

Similarly, gut-derived polyamines (putrescine, spermidine, and spermine)^8^ and water-soluble vitamins^9^ are established through a balance of division of labor and competition between microbes, as well as between microbes and the host. In particular, vitamin B12 (cobalamins/cobamides) is a notable example of cross-feeding; while only limited bacteria can perform de novo synthesis, sharing, modification, and recycling occur through the structural diversity of pathway intermediates and cobamides.^10^

Traditionally, most fecal metabolites have been evaluated as total amounts. However, since fecal concentrations are simultaneously influenced by microbial production, intracellular retention, consumption by other species, and host absorption, it is fundamentally difficult to distinguish producers from consumers (users) based on a single total measurement. This creates an interpretive bottleneck where changes in metabolic potential suggested by functional gene analysis do not necessarily align with changes in measured metabolite levels.

In this study, we present an operational framework to differentiate between extracellular-enriched and cell-associated (intracellular) pools within the same sample by quantifying gut microbiota-derived metabolites (SCFAs, polyamines, and water-soluble vitamins) in both undisrupted (a fraction approximating the extracellular pool) and post-disruption (a fraction approximating the total pool) fecal samples. SCFAs and polyamines are quantified as extracellular fractions, while vitamins are measured in both the extracellular fraction and the intracellular component released through disruption. By integrating microbial composition, functional genes, and metabolite levels, we aim to identify major candidate producers and users of metabolites in the gut and clarify the contributions of the underlying cross-feeding networks. It should be noted that the undisrupted fraction is an approximation of the extracellular pool, and leakage due to natural lysis or sample processing cannot be entirely excluded.

## Methods

### Ethics statement

All studies were approved by the Ethical Review Committees (ERC) of the Nagoya University Graduate School of Medicine (Approval No: 2016-0151), Iwate Medical University (H28-123), and Okayama Kyokuto Hospital (kyoIR-2016002). Written informed consent was obtained from all healthy participants.

#### Subjects and sample collection

Fecal samples were collected from 63 healthy individuals. Samples of 10 g or more were collected at the participants' homes and transported to Nagoya University maintained at 0 °C with cooling agents in insulated jars. The samples were freeze-dried using a lyophilizer (FDU-2110, EYELA, Shanghai, China). To eliminate the potential for two-fold variations in metabolite concentrations depending on the sampling site,^11^ the freeze-dried feces were disrupted into a fine powder to ensure homogenization.

#### Metagenomic analysis

DNA was extracted from 20 mg of freeze-dried (FD) feces using the QIAamp PowerFecal DNA Kit (QIAGEN, Hilden, Germany). The protocol was modified to replace vortexing with mechanical disruption using a FastPrep-24 5 G (MP Biomedicals) and Lysing Matrix E Beads (MP Biomedicals, Irvine, CA, USA) at 6.0 m/s for 60 seconds (three cycles). This intense mechanical disruption condition is identical to the protocol we used for metagenomic DNA extraction, which has been established as highly effective for breaking the resilient cell walls of diverse gut microbiota to release intracellular contents.^12^ ^,^ ^13^

Sequencing libraries were prepared using the NEBNext Ultra DNA Library Prep Kit for Illumina (NEB, Ipswich, MA, USA) following the manufacturer's instructions and tagged with index codes. Library quality was assessed via TapeStation (Agilent, Santa Clara, CA, USA). Metagenomic sequencing was performed on a HiSeq 2500 (Illumina, San Diego, CA, USA) with 2 × 150 bp paired-end reads, achieving approximately 3 Gbp per sample.

The raw shotgun reads were quality-controlled using fastp (v0.23.4), and human genome (GRCh38) sequences were removed using bowtie2 (v2.5.0) with default parameters. Subsequently, HUMAnN 3.6 was used to calculate Copies Per Million (CPM) for each EC number, and MetaPhlAn 4 was employed to determine the relative abundance of bacterial genera and species.^14^ ^,^ ^15^

#### Metabolite quantification

Short-chain fatty acids (SCFAs), polyamines, and water-soluble vitamins were quantified from freeze-dried (FD) fecal powder. The process details for preparing the FD form are described in our previous study.^11^

Short-Chain Fatty Acids (SCFAs): 20 mg of FD feces were mixed with 1000 µL of 5 mmol/L NaOH and 300 µL of water, followed by vigorous shaking with zirconia beads for 10 minutes. After centrifugation, samples were derivatized using 2-methyl-1-propanol, pyridine, and isobutyl chloroformate. The organic phase was then analyzed using gas chromatography-mass spectrometry (GC-MS).^11^

Water-Soluble Vitamins: The FD fecal samples were dispensed in 2 mL tubes at 20 mg. 1 mL of water was added to 20 mg of feces. The mixture was homogenized for 10 minutes using a Micro Mixer E-26 (TAITEC, Saitama, Japan), manually shaken vertically 30 times, and homogenized again for 10 minutes. The mixture was then centrifuged at 12,000 rpm for 5 minutes. 200 μL of the supernatant was pipetted into a polypropylene tube (each tube volume was 2 mL), and 300 μL of 2% formic acid were added. Then, 50 μL each of I.S. solutions (100 mg/L folic acid-d₄ and 100 mg/L biotin-d₄) were added to 500 μL of the diluted fecal samples. After gentle shaking, the prepared fecal sample was subjected to an SPE procedure using a centrifuge. Preconditioning of the ABN cartridge was achieved by conditioning with 500 μL of methanol, followed by equilibrating with 500 μL of 0.1% formic acid. Then, 400 μL of the prepared fecal sample was loaded into the ABN cartridge. After passing the sample through the ABN cartridge, a washing procedure was performed with 1 mL of 0.2% formic acid. The target compounds were eluted with 1 mL of 50% methanol. Finally, the eluent was analyzed with LC-MS/MS. Disruption was performed using Lysing Matrix E Beads at 6.0 m/s for 60 seconds (three cycles).

#### Validation of the mechanical crash/lysis procedure

To validate the efficacy of the mechanical crash/lysis procedure and to assess whether the procedure affects residual enzymatic activity or vitamin recovery, independent validation experiments were performed using unrelated freeze-dried human stool samples processed under the same conditions as the study samples.

For assessment of bacterial disruption, 20 mg FD fecal powder were centrifuged in water (12,000 rpm, 5 minutes) to separate the uncrushed supernatant. DNA was then extracted from the pellet (QIAamp PowerFecal DNA Kit) using the same bead-beating parameters as the primary analysis (6.0 m/s, 60 seconds, 3 cycles; FastPrep-24 5 G). 16S rRNA gene levels were quantified by qPCR on a LightCycler 480 using TB Green Premix Ex Taq II and EMP (The Earth Microbiome Project) universal primers (FWD: 5′-GTGYCAGCMGCCGCGGTAA-3′; REV: 5′-GGACTACNVGGGTWTCTAAT-3′). Bacterial DNA was almost undetectable in the pre-disruption extracellular supernatant, whereas sufficient bacterial DNA was recovered after mechanical disruption, indicating effective bacterial cell disruption.

To assess whether the crash/lysis procedure adversely affects residual enzymatic activity, LDH activity was measured before and after disruption using LDH Assay Kit Colorimetric (abcam Cambridge, UK) according to the manufacturer's instructions. LDH activity was detectable before disruption. LDH activity did not increase upon disruption and instead exhibited a slight decrease after the crash/lysis procedure. This indicates that the mechanical process partially affects the enzyme and that measuring the intracellular release of bacterial LDH is difficult in this fecal matrix.

To assess the stability and recovery of the target low-molecular-weight analytes during the crash/lysis procedure, spike-recovery experiments were performed using vitamin standards.

Fractionation and disruption effect: to distinguish between extracellular and intracellular contributions, vitamins were measured in both undisrupted samples (approximating the extracellular pool) and disrupted samples (approximating the total pool). To account for potential thermal degradation or loss during mechanical disruption, recovery rates were determined through a spike-and-recovery test (adding standards before and after disruption), and measured values were corrected accordingly. The vitamin B stability for mechanical disruption (degradation rate) was determined by absolute recovery test. The standard solution mixture of vitamins was spiked at two different points in the measurement procedure, a spike into FD before mechanical disruption (i) and a spike into FD after mechanical disruption (ii). The recoveries of the measurement procedure were calculated by comparing analyte peak area. The degradation rate of vitamins by mechanical disruption was calculated as the mean area of the results for (ii) and those for (i).

Polyamines: Putrescine, spermidine, and spermine were measured by LC-MS/MS using a modified version of the method by Xiong and Zhai (2016). Samples were derivatized with Fmoc solution (50 mmol/L in acetonitrile) after extraction and then subjected to analysis.

#### Statistical analysis

For functional genes (EC numbers) and bacterial relative abundance at the genus level, only features with a zero-value frequency of 40% or less were included in the analysis. Zero values were replaced with half of the minimum detected value for each feature, followed by Centered Log-Ratio (CLR) transformation. SCFAs and polyamines were log-transformed after similar zero-value substitution.

Vitamins were analyzed as total (T) after disruption and extra (E) before grinding. After replacing zero values with half the minimum value for each vitamin (c), data were log-transformed. The indicator for intracellular contribution, log(T + c) − log(E + c), was calculated. Feature selection for network analysis was performed using Elastic Net (linear model) and Random Forest with Boruta (nonlinear model) to robustly capture both linear and complex nonlinear relationships while mitigating multicollinearity and overfitting. Features selected in at least three out of five cross-validation folds were utilized for correlation analysis.

The effect of mechanical disruption was estimated using a linear mixed model with subject-specific random intercepts, with multiple testing corrected using the false discovery rate (FDR). Partial Spearman correlation was used to adjust for fecal moisture content, and the *P*-values from all correlation analyzes were adjusted for multiple testing using the false discovery rate (FDR) method (Benjamini‒Hochberg procedure). The correlation threshold was set at |*r*| > 0.3 as an operational cutoff to filter out the background noise commonly observed in highly variable microbiome datasets, thereby focusing on biologically meaningful, moderate-to-strong associations. Significant correlations (FDR-adjusted *P* < 0.05 and |*r*| > 0.3) between metabolites and EC numbers were visualized using KEGG mapper to identify their positions within metabolic pathway maps.

## Results

### Clinical characteristics of subjects

The study cohort consisted of 63 healthy individuals (31 males and 32 females) with a mean age of 66.9 ± 9.8 y and a mean BMI of 22.6 ± 3.1 kg/m^2^. The average defecation frequency was 8.2 ± 4.6 times per week, and the dry/wet stool weight ratio was 0.76 ± 0.08.

Regarding dietary habits, 32 participants consumed milk daily, while 19 reported no milk intake. Univariate correlation analysis revealed that the abundance of *Bifidobacterium* was higher in females and frequent milk consumers but tended to decrease with age. However, these findings should be interpreted with caution as they are based on univariate analysis, and there is a potential confounding effect between gender and milk intake behavior (e.g., a disproportionately higher number of females among frequent milk consumers). Furthermore, fecal moisture content was positively associated with the abundance of short-chain fatty acid (SCFA)-producing bacteria. In contrast, the genus *Alistipes* was more abundant in subjects with lower fecal moisture content and lower defecation frequency (Supplemental Figure 2). The mean relative abundance and prevalence (detection rate within our study cohort) of these key taxonomic groups discussed below are summarized in the Supplementary Data (genus_exist_rate.csv).

### Validation of bacterial disruption and analyte stability

We performed validation experiments using unrelated freeze-dried stool samples processed in the same manner as the study samples. Bacterial DNA/16S rRNA gene recovery was used to assess bacterial disruption. DNA was nearly absent from the extracellular supernatant before disruption, whereas sufficient bacterial DNA was recovered after the crash/lysis procedure. This supports effective disruption of bacterial cells by the protocol (supplement Table 1A).

LDH activity was also measured before and after the disruption/lysis procedure. LDH activity did not increase and showed a slight decrease after disruption. This demonstrates that the mechanical disruption partially affects the enzyme, making it difficult to measure intracellular bacterial LDH or use it as a quantitative marker of bacterial lysis in these fecal samples (supplement Figure 1B, Table1B).

Finally, vitamin standard spike-recovery experiments showed that the target low-molecular-weight analytes are partially affected by the disruption procedure. To maintain quantitative accuracy, a correction was performed for the vitamin measurements to account for the impact of the disruption process (supplementary Figure 1C).

### Intracellular and extracellular dynamics of water-soluble vitamins via mechanical disruption

**Figure 1.**
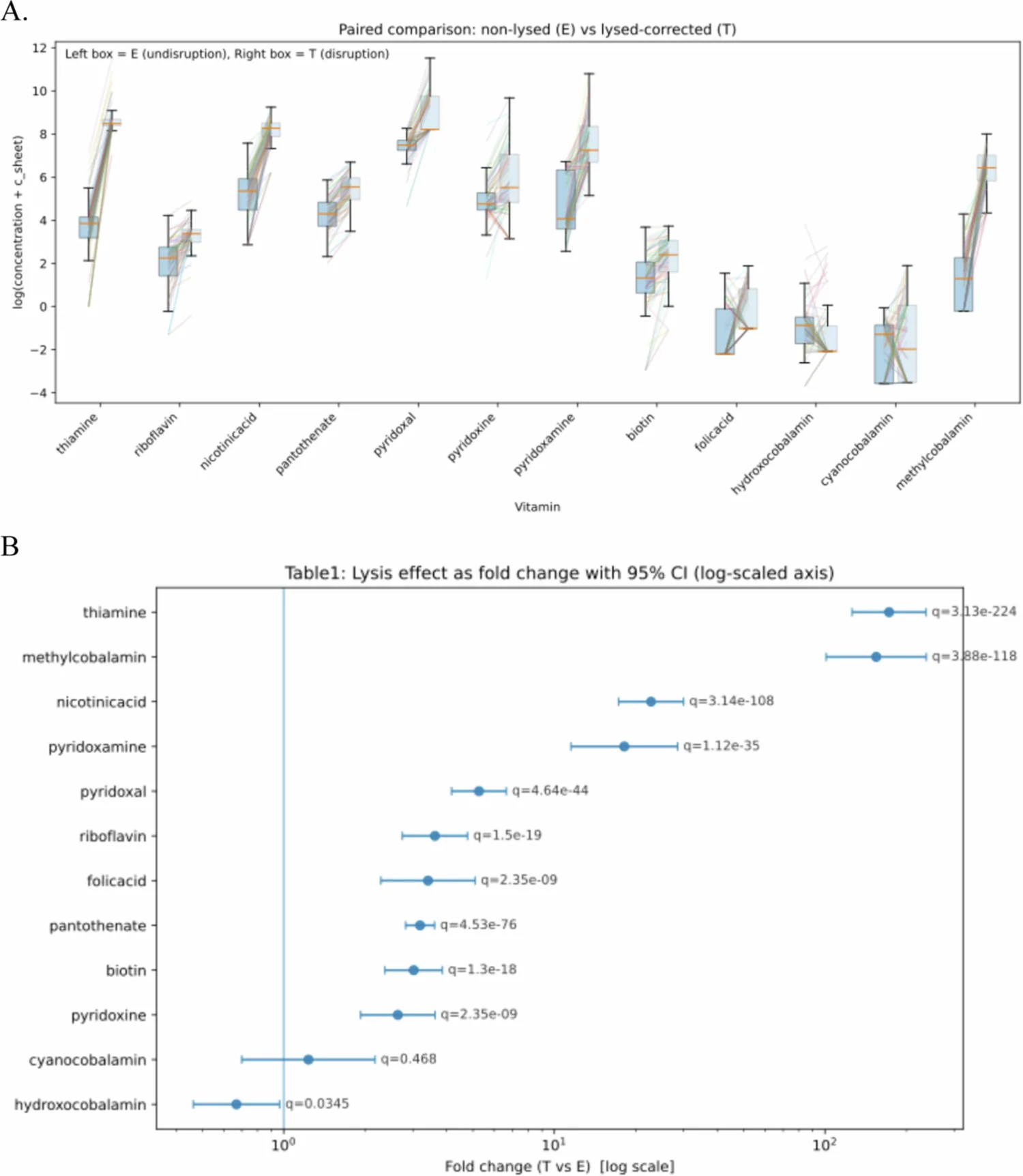
Fractionation of fecal vitamins and the effect of mechanical disruption. The abundance of fecal vitamins varies significantly depending on whether the samples are subjected to mechanical disruption (bead-beating). Vitamins were analyzed by defining the disrupted samples as “total (T)” and the undisrupted samples as “extracellular (E)”. Zero values were replaced with half of the minimum detected value for each vitamin, followed by log-transformation. A: The y-axis represents the log-transformed values for undisrupted and disrupted samples, visualized using box plots. B: The effect of disruption is shown on the x-axis as a fold change. • Adenosylcobalamin is excluded from this figure as it was not detectable in the undisrupted (extracellular) fraction. The vitamins are categorized into those whose abundance increased by 10-fold or more after disruption and those with less than a 10-fold increase.

To differentiate between the extracellular and intracellular contributions of fecal vitamins, we compared measured values from undisrupted samples (approximating the soluble/extracellular fraction) and disrupted samples (approximating the total pool including the intracellular fraction) from the same subjects. The impact of mechanical disruption varied significantly across different vitamins (Figure 1A and B).

**Figure 2.**
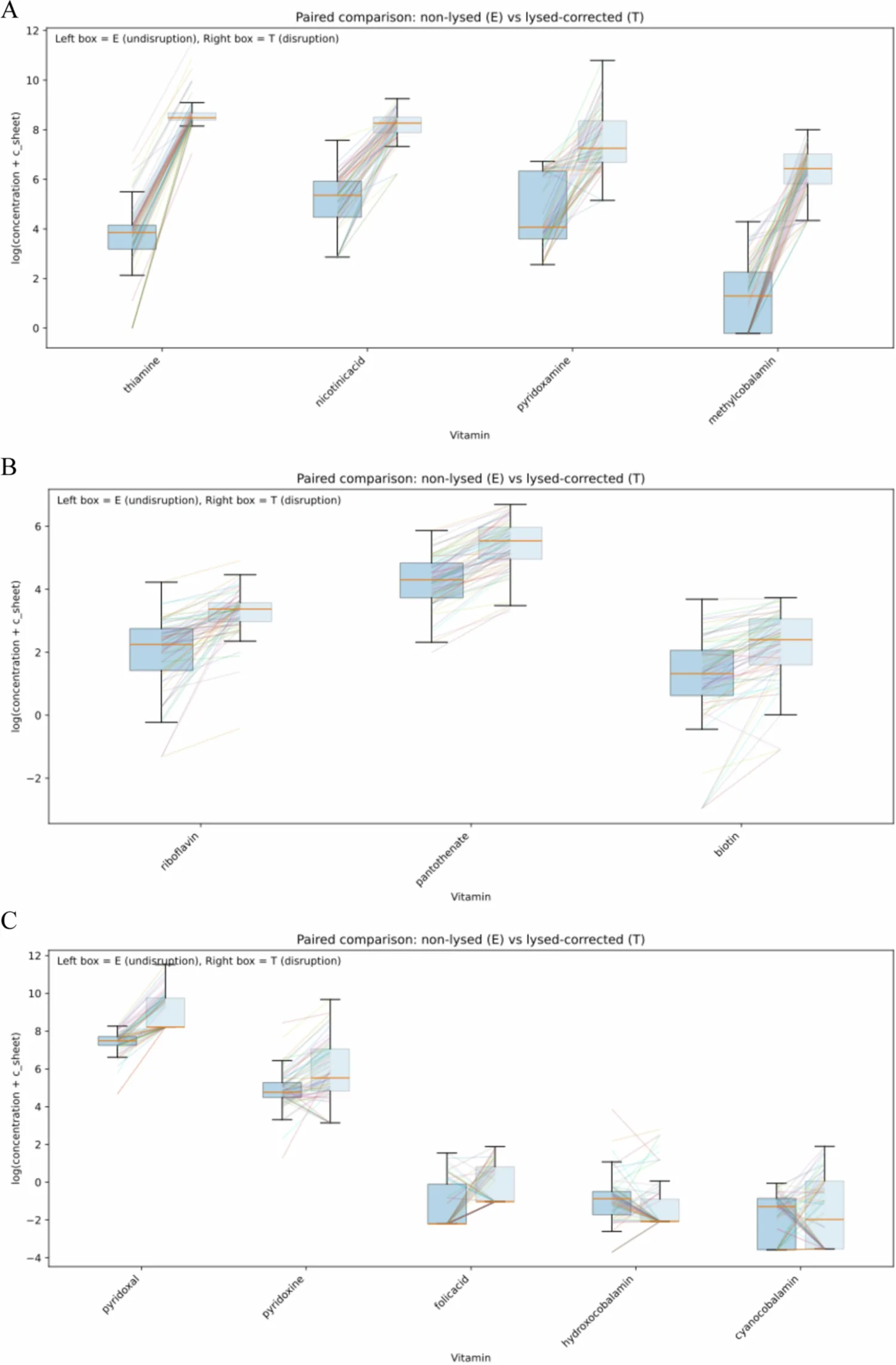
Differential impact of disruption on vitamin recovery and classification. The horizontal axis represents undisrupted and disrupted samples, and the vertical axis indicates: the log-transformed abundance of vitamins. A: Vitamins with a high intracellular contribution, where disruption increased detected levels by 10-fold or more. B: Vitamins with a relatively lower intracellular contribution, where disruption increased detected levels by approximately 1.5-fold (50% increase). C: Vitamins exhibiting high rates of zero-values or decreased detection after disruption, possibly due to thermal degradation or other unknown mechanisms (e.g., folic acid). Based on these behaviors, vitamins in groups A and B were classified as Type 1 (predominantly intracellular) and Type 2 (higher extracellular presence), respectively.

Notably, adenosylcobalamin (a vitamin B12 coenzyme) was undetectable in undisrupted samples and was identified only after disruption. Several other vitamins, including methylcobalamin, thiamine (B1), nicotinate (B3), and pyridoxamine (B6), showed undisrupted levels that were less than 10% of their total post-disruption levels (Figure 2A). This observation aligns with previous reports suggesting that these vitamins are predominantly utilized and tightly retained as intracellular cofactors by gut microbes, thereby indicating a high intracellular contribution^9^ ^,^ ^16^. These vitamins were categorized as Type 1 (predominantly intracellular), suggesting they are primarily retained within microbial cells or rapidly consumed upon release.

In contrast, for pantothenate (B5), riboflavin (B2), and biotin (B7), undisrupted values accounted for approximately 40% of the total measured post-disruption, indicating a relatively higher ratio of extracellular presence (Figure 2B). These were categorized as Type 2 (extracellularly available).

**Figure 3.**
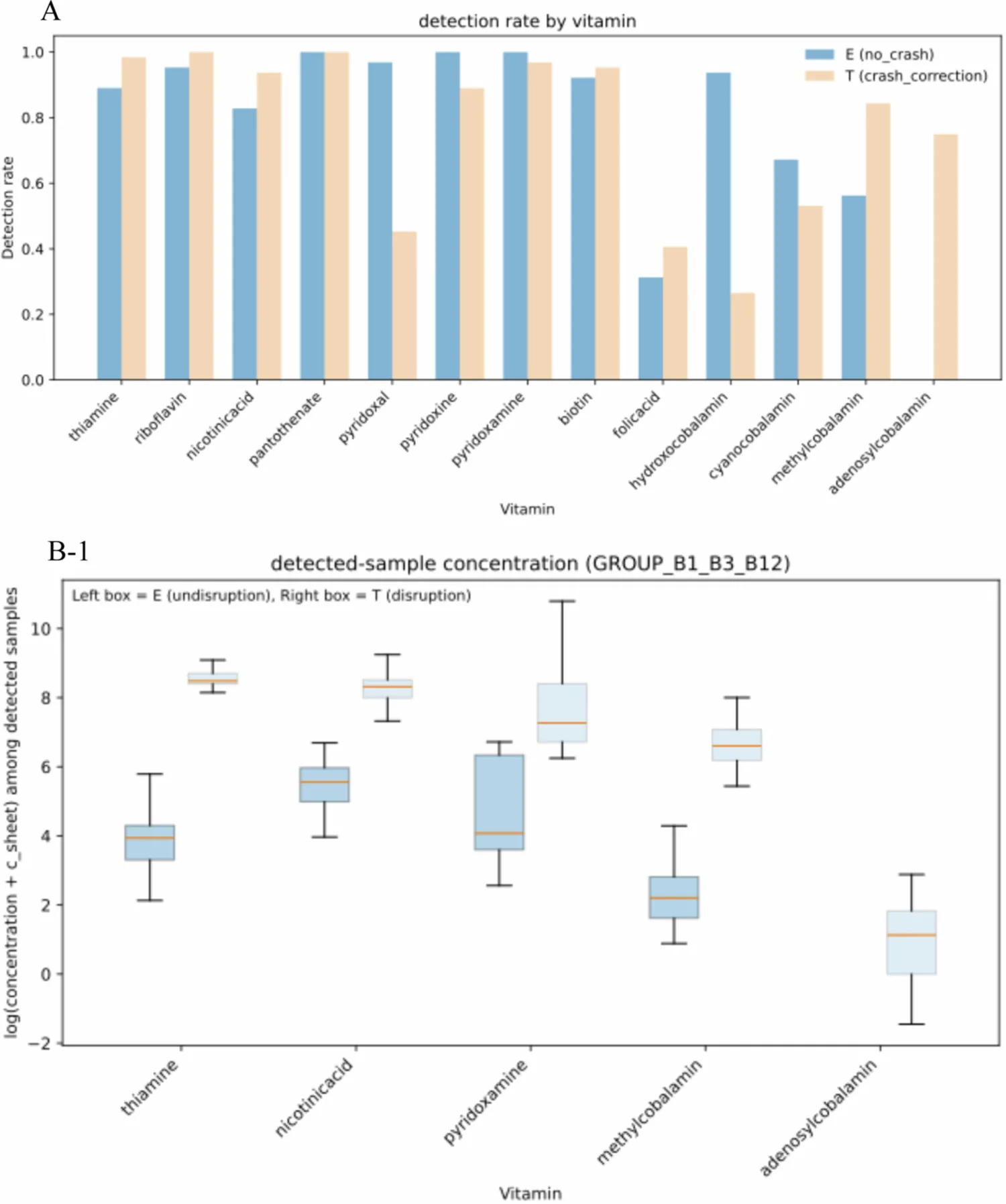
Detection ratios and disruption effects via linear mixed models. A: Comparison of detection ratios between undisrupted and disrupted samples. The x-axis represents the measured vitamins. Pyridoxal, folic acid, and hydroxycobalamin frequently fell below the limit of detection after disruption. B: Box plots representing the measured values (excluding undetectable samples). To clearly visualize the changes observed for each variable, individual graphs are presented for each vitamin. The vitamins are categorized into three groups based on their response to mechanical disruption: Type 1 (predominantly intracellular, showing substantial increases) (Figure 3 B-1) , Type 2 (higher extracellular presence, showing moderate increases) (Figure 3 B-2) , and vitamins exhibiting decreased levels or reduced detection after disruption (Figure 3 B-3) . A linear mixed model using subject-specific random intercepts confirmed significant increases after disruption for all vitamins except cyanocobalamin.

Certain vitamins, such as pyridoxal, folic acid, and hydroxycobalamin, exhibited inconsistent behaviors, including decreased detection rates or concentrations following disruption, possibly due to thermal degradation or other unknown mechanisms (Figure 2C). However, linear mixed model analysis confirmed that, with the exception of cyanocobalamin, mechanical disruption significantly increased the measured abundance of most vitamins across the cohort (Figure 3B-1,2,3).

### Associations between SCFAs, polyamines, and gut microbiota

**Figure 4.**
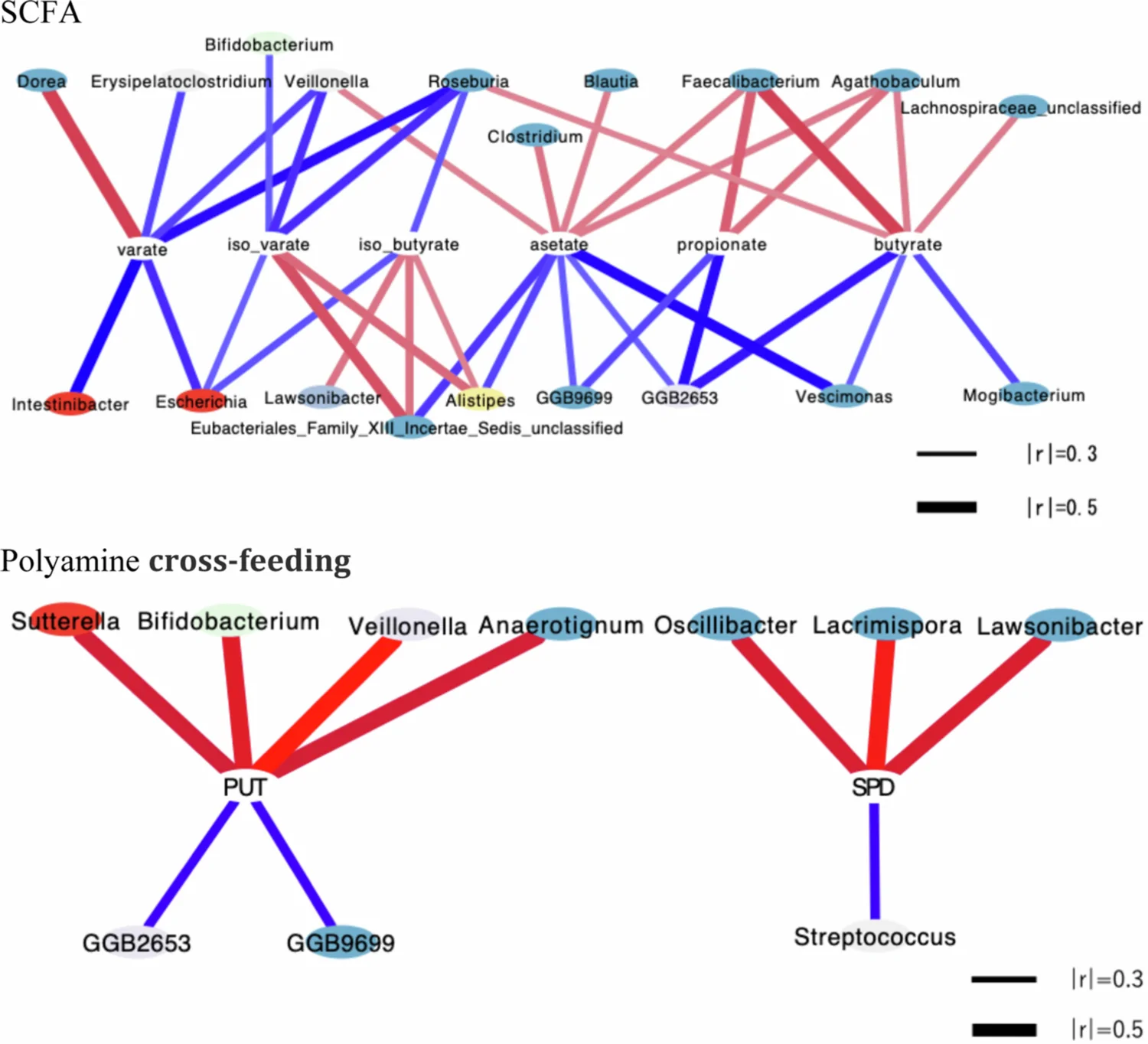
Correlation network between SCFA/polyamines and gut bacteria. This network illustrates the relationships between metabolites (SCFAs and polyamines) and bacterial genera with correlations of |*r*| > 0.3. • Red lines represent positive correlations, and blue lines represent negative correlations, with line thickness indicating the strength of the correlation. • Node colors indicate taxonomic orders: *Bacteroidales* (yellow), *Eubacteriales* (blue), *Proteobacteria*(red) and *Bifidobacteriales* (green), and other (light purple). • Specific findings: A high correlation (*r* = 0.47) was observed between the butyrate-producer *Faecalibacterium* and butyrate levels. *Bifidobacterium* (*r* = 0.34) and *Veillonella* (*r* = 0.47) showed the highest positive correlation with putrescine.

Network analysis was performed for metabolites with correlations of |*r*| > 0.3 with bacterial genera. High positive correlations were observed between valerate and Dorea, iso-valerate and *Eubacteriales_Family_XIII,* and butyrate and *Faecalibacterium* (Figure 4A). Among polyamines, few bacterial genera showed strong correlations; however, *Bifidobacterium* (*r* = 0.34) and *Veillonella* (*r* = 0.47) exhibited the highest positive correlation with putrescine (Figure 4B).

### Associations between vitamins and gut microbiota

The relationship between vitamins and gut bacteria was analyzed separately for Type 1 and Type 2 vitamins.

**Figure 5.**
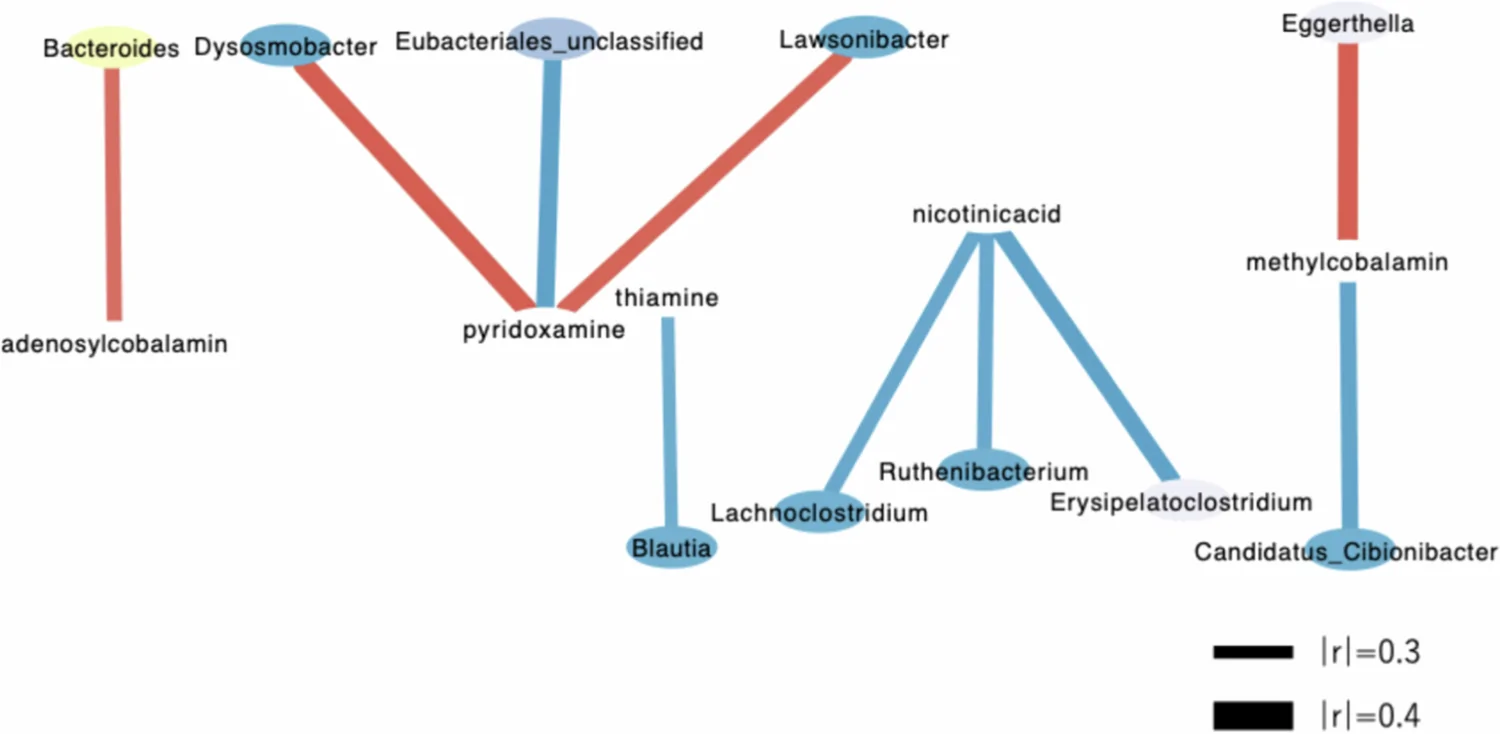
Correlation between Type 1 Vitamins and gut bacteria. This figure shows the relationship between Type 1 vitamins (adenosylcobalamin, methylcobalamin, thiamine, nicotinate, and pyridoxamine)—which have high intracellular contributions—and bacterial abundance. The analysis focuses on disrupted samples (total pool) as no significant correlations (|*r*| > 0.3) were observed in the undisrupted (extracellular) fraction for Type 1 vitamins. • Line thickness and colors follow the same conventions as Figure 4.

**Figure 6.**
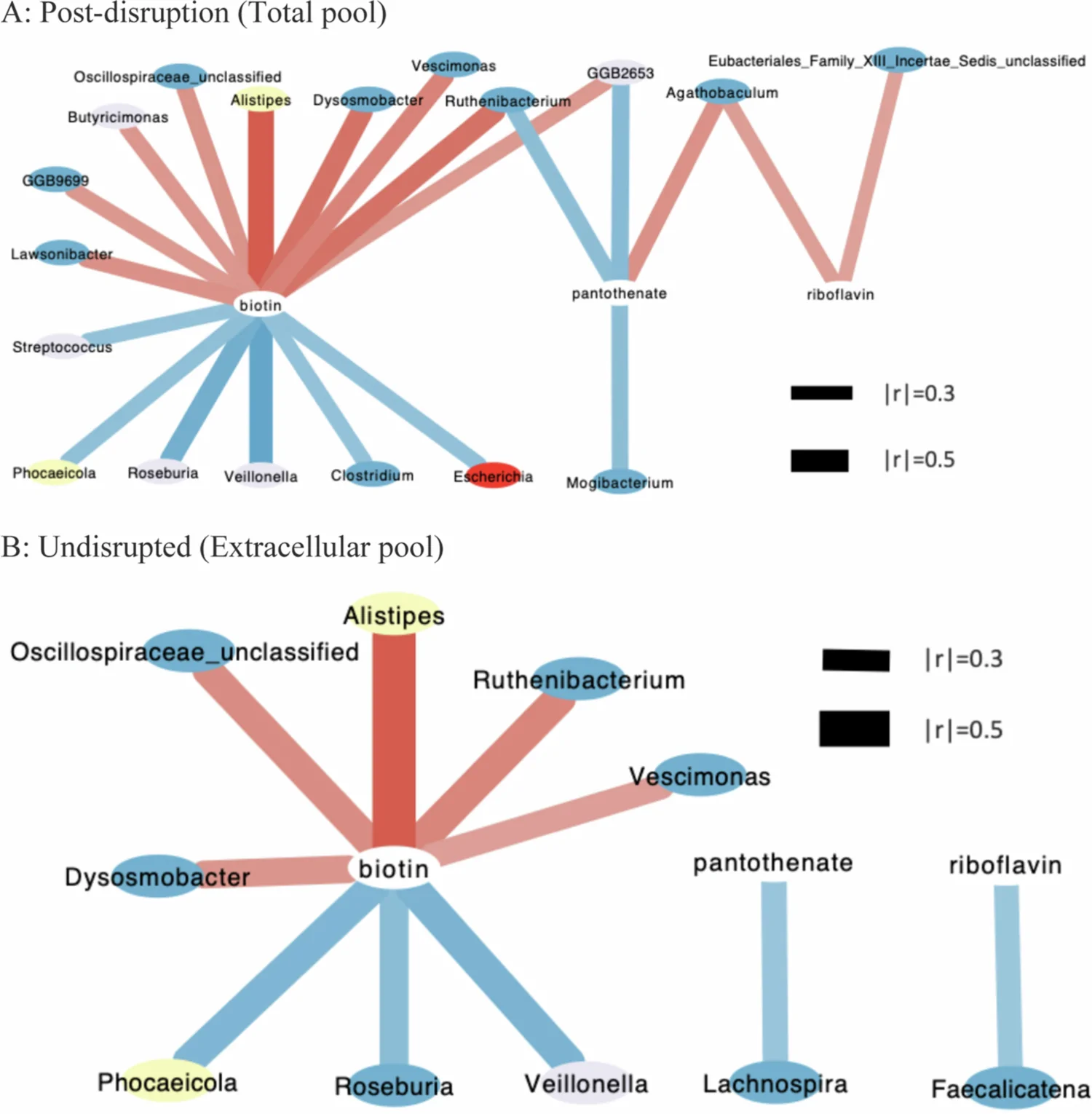
Correlation between Type 2 Vitamins and gut bacteria in different fractions. The relationship between Type 2 vitamins (riboflavin, pantothenate, and biotin)—which are considered to be secreted or present extracellularly—and bacterial genera is shown. Notably, *Alistipes* demonstrated a strong positive correlation with biotin in both the disrupted (*r* = 0.56) and undisrupted (*r* = 0.56) fractions.

- Type 1 Vitamins (total pool): positive correlations were identified between *Eggerthella* and methylcobalamin, *Lawsonibacter* and *Dysosmobacter* and pyridoxamine, and *Bacteroides* and both adenosylcobalamin and nicotinate (*r* > 0.3) (Figure 5). Notably, *Blautia* showed a significant negative correlation with thiamine (*r* = −0.31). In the undisrupted (extracellular) fraction, no bacterial genera achieved a correlation of |*r*| > 0.3 with Type 1 vitamins.
- Type 2 Vitamins (extracellular and total pools): biotin showed a strong positive correlation with *Alistipes* in both the disrupted (*r* = 0.56) and undisrupted (*r* = 0.56) fractions (Figure 6). Negative correlations for biotin were observed with *Roseburia*, *Veillonella*, and *Phocaeicola*. Additionally, Agathobacter correlated positively with pantothenate and riboflavin. The trend remained unchanged even in the partial Spearman correlation adjusted for moisture content.

### Species-level associations and intra-genus mutual exclusivity

Following our genus-level observations, we extended our correlation analysis to the species level (Supplementary Figure 4: species_type1 and species_type2). Surprisingly, the strong metabolite-microbe associations observed at the genus level, where multiple species exist, were largely attenuated at the species level. To investigate the underlying ecological dynamics, we analyzed the co-occurrence patterns of species within key genera, specifically focusing on *Alistipes* and *Blautia*. A contingency summary analysis (*α* = 0.05) revealed a high frequency of significant mutually exclusive interactions within the same genus: 40.0% (8 out of 20 pairs) for *Alistipes* species and 44.4% (32 out of 72 pairs) for *Blautia* species. In striking contrast, the frequency of significant interactions between different genera (*Alistipes* vs. *Blautia*) was markedly lower at only 17.8% (16 out of 90 pairs). These detailed species-level interaction patterns and statistical summaries are provided in the Supplementary Data(co-occurrence.tsv).

### Functional genes and metabolic pathways

To identify functional genetic markers, we extracted EC numbers associated with metabolites using Elastic Net and Boruta algorithms. Regarding polyamines, spermine was positively correlated with agmatine deiminase (EC:3.5.3.12) and ornithine cyclodeaminase (EC:4.3.1.12). For SCFAs, butyrate showed a positive correlation with acetyl-CoA C-acetyltransferase (EC:2.3.1.9).

In vitamin metabolism, nicotinate (Type 1) correlated with nicotinamidase (EC:3.5.1.19), and biotin (Type 2) correlated with 6-carboxyhexanoate-CoA ligase (EC:6.2.1.14). However, these functional genes generally did not align with the core metabolic pathways of the corresponding metabolites as defined in standard KEGG maps.

### Correlation between vitamins and other fecal metabolites

**Figure 7.**
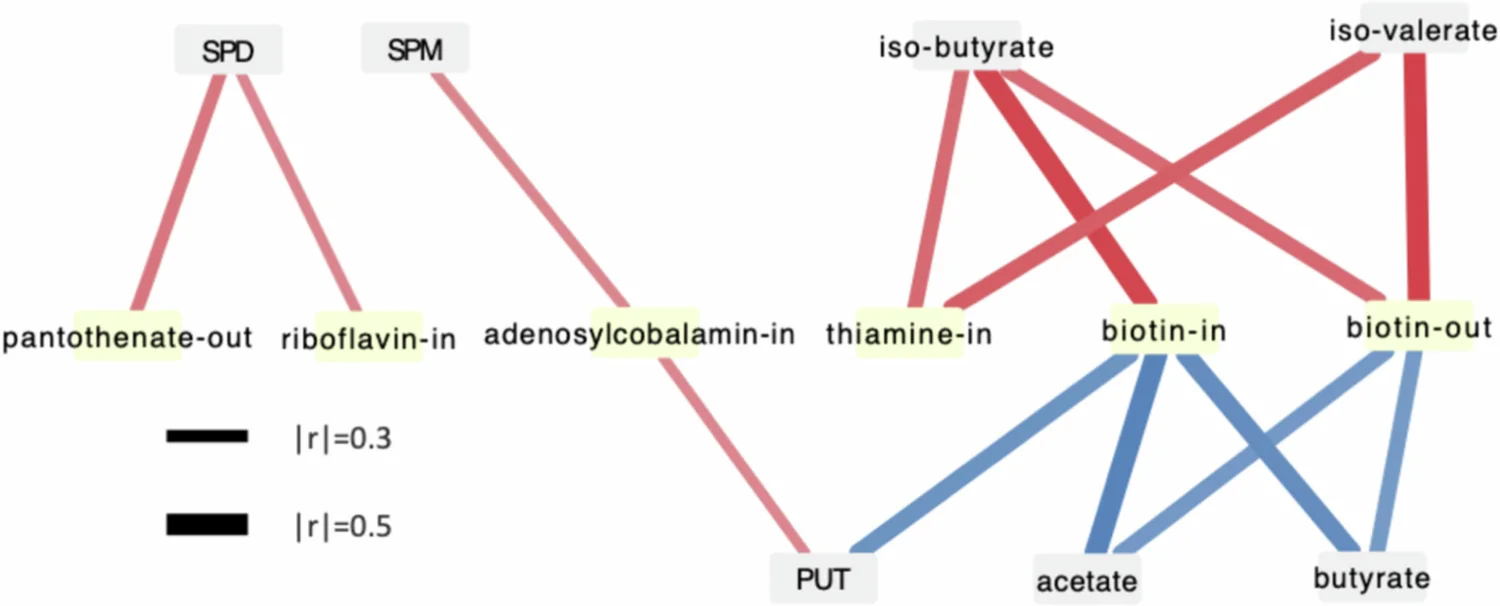
Association between Type 2 Vitamins and other fecal metabolites. This network displays correlations (|*r*| > 0.3) between Type 2 vitamins and other metabolites (SCFAs, BCFAs, and polyamines). • Metabolites are represented by gray rectangles, and vitamins by yellow rectangles. • Biotin showed positive correlations with BCFAs (branched-chain fatty acids) and negative correlations with SCFAs.

We further analyzed the associations between vitamins and other metabolites (SCFAs, BCFAs, and polyamines), adjusting for fecal moisture content. Biotin levels were positively correlated with branched-chain fatty acids (BCFAs), such as iso-butyrate and iso-valerate, but negatively correlated with SCFAs, such as acetate and butyrate (Figure 7). When further adjusted for fecal pH, the separation between the BCFA-associated cluster and the SCFA-associated cluster became more distinct (supplemental Fig.3). Detailed network data can be found in the supplementary_data (Figures 4–7.csv data).

## Discussion

### Main findings and significance

In this study, we addressed the inherent variability of fecal metabolite concentrations by homogenizing large-volume fecal samples (over 10 g) through lyophilization and powderization. The primary novelty of our approach lies in the fractionation of fecal metabolites into undisrupted (approximating the extracellular pool) and disrupted (approximating the total pool) samples. This operational methodology allows for the differentiation between predominantly extracellular and cell-associated metabolite pools, overcoming a major confounding factor in conventional “total amount” metabolomics. Our findings reveal that the impact of mechanical disruption varies significantly across metabolites, providing a new analytical axis to resolve the discrepancies between metagenomic potential (functional genes) and metabolomic reality.

### Interpretation of short-chain fatty acid (SCFA) and polyamine networks

Short-chain fatty acids (SCFAs) are the primary metabolic end-products from which gut microbiota derive energy. Because organic acids can exhibit cellular toxicity, they are generally released into the extracellular environment. Our network analysis identified a strong positive correlation (*r *> 0.4) between butyrate and *Faecalibacterium*, which is highly consistent with its established role as a major butyrate producer.

Regarding other metabolites, *Alistipes* showed positive associations with acetate and branched-chain fatty acids (BCFAs). The observed negative correlation between *Alistipes* and *Roseburia* may represent an apparent association reflecting environmental factors, such as fecal pH or moisture content. Specifically, *Roseburia* tends to predominate in weakly acidic environments,^10^ while BCFAs often increase under conditions where proteolytic fermentation is dominant.^17^

For polyamines, the arginine → agmatine → putrescine pathway is often treated as a major route for putrescine biosynthesis (representative genes: speA → speB, or aguA/aguB).^18^ ^,^ ^19^ This strategy is consistent with the use of arginine metabolism under acid stress, and the positive correlation between putrescine and *Bifidobacterium* may reflect an association with a more acidic fecal environment driven by lactic acid production by *Bifidobacterium*.

### Fractionation-resolved dynamics of water-soluble vitamins

In the water-soluble vitamin panel, many analytes showed marked increases in total abundance after disruption, and the observation that adenosylcobalamin was essentially undetectable in the undisrupted fraction but became detectable only after disruption was particularly emblematic. This pattern is consistent with the fact that the vitamin is not predominantly present as a free metabolite; rather, it functions mainly as an enzyme-bound cofactor (e.g. associated with methylmalonyl-CoA mutase) within the cell. Such protein-bound forms may not be efficiently extracted without mechanical disruption (e.g. bead-beating) that physically breaks cells and protein complexes. In addition, de novo cobalamin biosynthesis is an exceptionally energy-intensive process requiring ~30 enzymatic steps, and only a limited subset of gut microbes can synthesize vitamin B12. In contrast, demand for cobalamin is high across the community. Therefore, even if cobalamin is transiently released into the extracellular milieu through spontaneous lysis or secretion, it may be rapidly scavenged by surrounding microbes via high-affinity transporters.^20^ The lack of detection in the undisrupted fraction is also compatible with the possibility that the completed cofactor is primarily consumed and/or retained intracellularly. Furthermore, because *Bacteroides* species carry *btuB* and may exhibit strong B12 uptake capacity,^21^ ^,^ ^22^ interpretation of correlations should explicitly consider the contribution of uptake as well as production. Collectively, we propose that the failure of adenosylcobalamin to accumulate extracellularly reflects the combined consequences of its “scarcity” and “essentiality,” which drive highly efficient intracellular sequestration and competitive community-wide reclamation.

**Figure 8.**
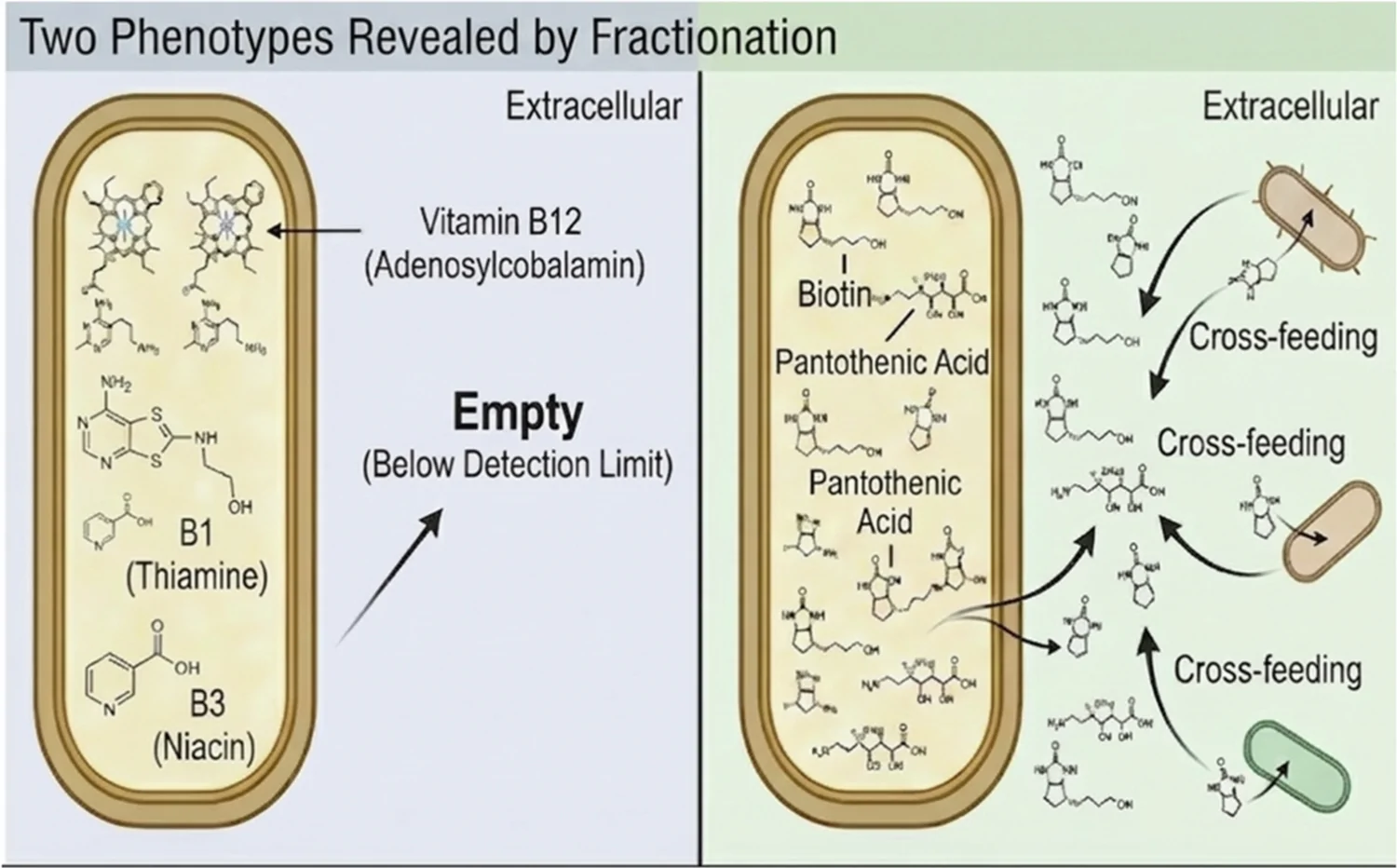
Vitamins exhibited different behaviors depending on fractionation. Adenosylcobalamin (vitamin B12 coenzyme) was detected only after cell disruption and was shown to be an “intracellular retention type (Type 1)” along with thiamine (B1), niacin (B3), etc., where the majority is retained within the bacterial cells. In contrast, biotin (B7) and pantothenic acid (B5) were classified as “extracellular release type (Type 2)” with a high rate of release outside the cell. De novo synthesis of cobalamin is an extremely energy-intensive process requiring approximately 30 enzymatic steps. Even if temporarily released extracellularly, it is immediately reabsorbed by high-affinity transporters present in surrounding bacteria, making extracellular measurement impossible.

In this study, at least for a subset of vitamins, the fraction-based measurements suggested two distinct behavioral phenotypes (Type 1 and 2). Type 1 comprised vitamins whose measured levels increased markedly after disruption, potentially reflecting a cell-associated intracellular pool retained by the gut microbiota. In contrast, Type 2 vitamins were relatively detectable even in the extracellular fraction, which may reflect a greater propensity for release, leakage, and/or sharing among microbes. As shown in Figure 2, Type 1 vitamins that exhibited high values only after disruption may be predominantly utilized intracellularly and, even if they transiently enter the extracellular milieu, rapidly consumed, thereby limiting their accumulation in the extracellular pool (Figure 8).

Niacin, classified as a Type 1 vitamin in this study, is an essential precursor of NAD/NADP and thereby indispensable for redox reactions and cofactor metabolism that supports homeostasis in both the host and microorganisms.^23^ While de novo niacin biosynthesis is absent in some lactic acid bacteria (e.g. *Lactobacillus plantarum*),^24^ many bacterial taxa are considered capable of synthesizing niacin endogenously.^25^ In our data, *Blautia* exhibited a negative correlation with thiamine. Thiamine (vitamin B1) can be costly to synthesize and is widely dependent on exogenous sources; accordingly, reduced biosynthetic capacity has been reported in the phylum Firmicutes, and *Faecalibacterium prausnitzii* is thought to lack thiamine biosynthesis.^26^

Given that *Blautia* often predominates in Japanese cohorts,^27^ it is plausible that certain *Blautia* lineages contribute significantly to thiamine dynamics. Mechanistically, fermentative metabolism and energy acquisition in obligate anaerobes such as *Blautia* spp. rely heavily on thiamine-dependent enzymes (e.g. pyruvate:ferredoxin oxidoreductase).^28^ Because this metabolic flux is large, sustained high-rate growth likely requires a continuous and sufficient supply of thiamine pyrophosphate (TPP), thereby increasing intracellular thiamine demand.^29^ The negative correlation observed between *Blautia* and thiamine (*r* = −0.31) provides a striking contrast to the *Alistipes*–biotin relationship. While *Alistipes* appears to act as a primary provider, our data suggest that *Blautia* functions as a significant consumer of thiamine. This is consistent with its classification as a Type 1 vitamin, where high intracellular demand for TPP likely accelerates the depletion of the extracellular thiamine pool. Thus, the 'intracellular-retained' phenotype of thiamine in this cohort may be driven by the vigorous fermentative activity of dominant taxa like *Blautia*. Two non-mutually exclusive factors may therefore contribute. First, an ecological mechanism: dominance of non-producers could reduce community-level thiamine production capacity. Second, a physiological mechanism: increased intracellular demand associated with vigorous fermentation may outpace supply, thereby accelerating depletion of environmental thiamine. However, for Type 1 vitamins in the present study, correlation coefficients were generally small, indicating that there are limits to explaining the findings solely by identifying specific taxa or by a simple producer–consumer dichotomy. Importantly, Type 1 readouts may be influenced not only by biological processes—such as cellular uptake and retention—but also by differences in microbial biomass and growth phase, as well as methodological factors including variability in lysis and extraction efficiency. In addition, intracellular pools are tightly regulated to maintain cellular homeostasis, and metabolite concentrations can vary nonlinearly with changes in microbial abundance (biomass). Consequently, correlation-based analyzes alone are insufficient to fully capture the underlying biology. Establishing causal mechanisms will therefore require complementary approaches, such as direct validation of production/consumption in culture-based systems, metatranscriptomic profiling, and flux assessment using stable isotope tracing.

In contrast, for biotin (Type 2), we observed a strong positive correlation with *Alistipes* and negative correlations with *Roseburia*, *Veillonella*, and *Phocaeicola*. A systematic metagenome-based assessment of B-vitamin biosynthetic potential has suggested that biotin biosynthesis capacity is relatively enriched in Bacteroidetes.^9^ Nevertheless, in healthy cohorts, quantitative evidence pinpointing which genera or species within Bacteroidetes are most strongly associated with fecal biotin levels remains limited. In the present study, we found a robust association between biotin and *Alistipes*, a genus within *Bacteroidetes*; to our knowledge, reports highlighting this specific relationship are scarce. *Alistipes*—previously referred to as *Bacteroides putredinis*—was reclassified in 2003.^30^ While the literature is extensive for organisms such as *Bacteroides thetaiotaomicron* and *Bacteroides fragilis*, functional studies focusing on *Alistipes* remain comparatively fewer. That said, vitamin biosynthetic potential in *Alistipes* (including biotin) has been examined,^16^ and KEGG pathway annotations assign biotin biosynthesis pathways to species such as *Alistipes putredinis* and *Alistipes shahii* (KEGG pathway maps).

**Figure 9.**
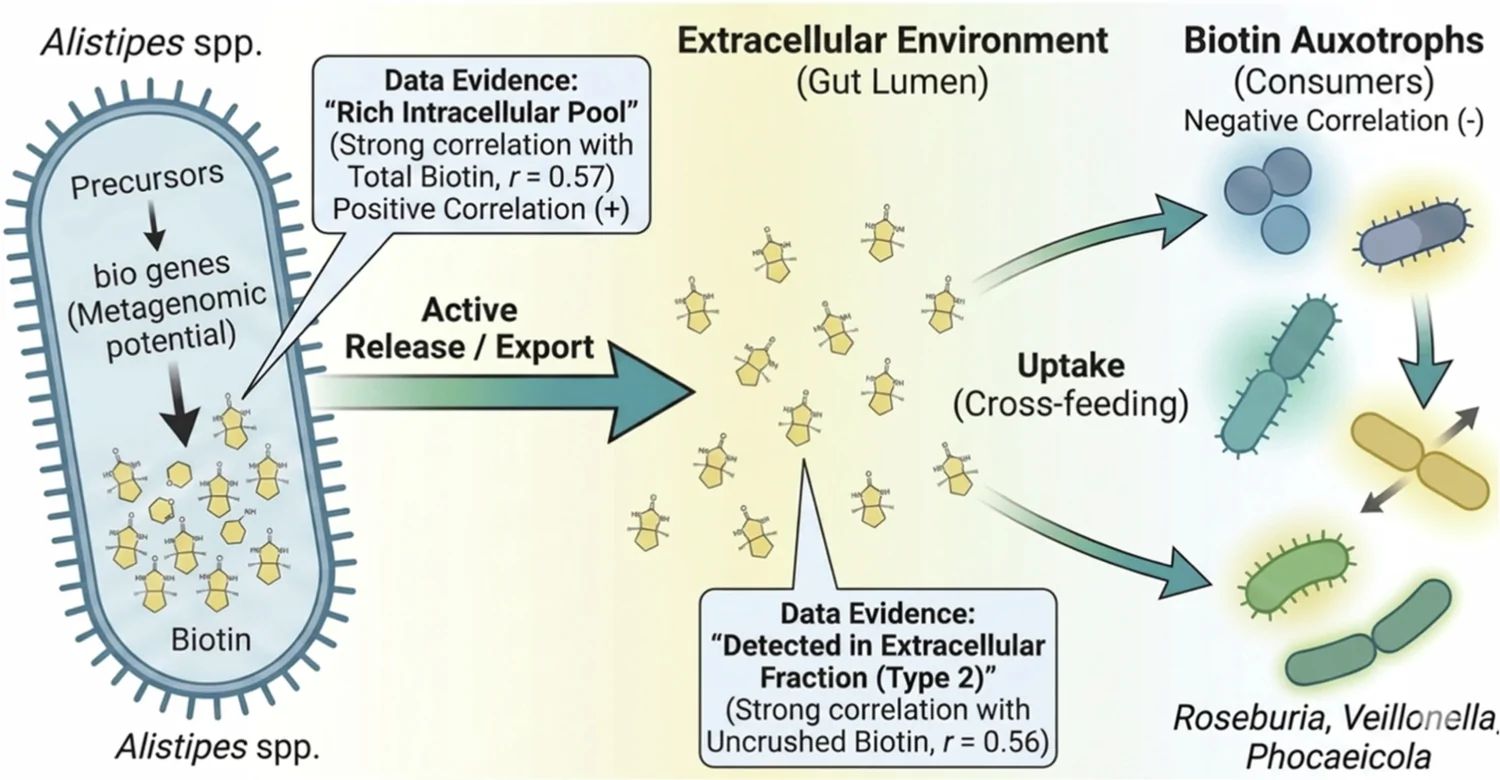
Biotin showed a strong positive correlation with *Alistipes* and a negative correlation with *Roseburia*, *Veillonella,* and *Phocaeicola*. The vitamin biosynthesis potential in *Alistipes* remains insufficiently studied. Furthermore, it was revealed that while these bacteria belong to the same Bacteroidetes order and possess biotin production capabilities, their roles vary depending on the environment.

Despite belonging to the same order, *Bacteroidales*, *Phocaeicola* showed a negative correlation with biotin, suggesting that its metabolic phenotype may shift toward relying on external biotin uptake when available. Similarly, the negative associations with *Roseburia* and *Veillonella* are consistent with their known ecological characteristics and metabolic dependencies (e.g., *Roseburia*'s reliance on biotin salvage and *Veillonella*'s use of biotin-dependent enzymes).^10^ ^,^ ^31^ These observations suggest that different taxa fulfill distinct roles as biotin providers or consumers in a context-dependent manner (Figure 9).

### Functional redundancy at the genus level

When extending our taxonomic resolution to the species level, we found that the significant correlations between vitamins and specific genera (e.g. *Alistipes* and biotin, *Blautia* and thiamine) became less apparent. Our subsequent co-occurrence analysis revealed robust mutually exclusive distributions among species within the same genus, but not between different genera. This pattern strongly suggests functional redundancy within these bacterial genera. In the gut ecosystem, closely related species within a genus often share core metabolic capabilities and compete for the same ecological niche. Consequently, different individuals may be colonized by distinct species of *Alistipes*, but the overall contribution of the *Alistipes* niche to the fecal biotin pool remains conserved. These findings underscore that, in the context of specific vitamin cross-feeding architectures, the genus level represents a highly coherent and ecologically relevant unit of analysis, effectively minimizing the analytical noise introduced by inter-individual variations in species-level colonization.

### Limitations and future directions

This study is based on cross-sectional data from healthy individuals; therefore, the observed correlations do not establish causality. To definitively prove actual metabolic fluxes and true mechanistic models of cross-feeding, direct experimental validation—such as stable isotope tracing or controlled *in vitro* co-culture experiments—is strictly required. In addition, fecal measurements represent an approximation of the intestinal luminal environment and are influenced by host absorption and transit time. Nevertheless, an important strength of our approach is that fractionation enabled within-sample comparisons between metabolites that are enriched in the extracellular environment and those that are primarily cell-associated. In particular, the apparent prominence of *Alistipes* in relation to biotin availability is a relationship that can be substantiated only through direct metabolite quantification, rather than from metagenomic potential alone. Moreover, vitamins classified as Type 2 may be more likely to participate in microbial cross-feeding and coexistence through extracellular release, and their potential links to disease states warrant further investigation.

## Supplementary Material

Supplement Fig 3.docx Supplement Fig 3.docx

Supplement Fig 2.docx Supplement Fig 2.docx

supplement Fig 4.docx supplement Fig 4.docx

Supplement Fig1.docx Supplement Fig1.docx

## Data Availability

Sequence data of our dataset are available at the DNA Data Bank of Japan (DDBJ) under the accession number of DRA016410. Data are available from corresponding author upon reasonable request from any qualified investigator.
