# Supplementary material for: Separating extracellular from intracellular fecal metabolites exposes cross-feeding architecture in the gut: exploring metabolite partitioning and ecological associations
Source: Gut Microbes. 2026 Aug 24;18(1):2719062. doi: 10.1080/19490976.2026.2719062 (PMC13505459; doi:10.1080/19490976.2026.2719062)
Supplement: Supplementary file 1 [file KGMI_A_2719062_SM8611.docx]

Supplement table1A

Representative qPCR data for validation of fecal fractionation.

| Fraction | Preparation / DNA Concentration | 16S rRNA gene (Cp value) | Interpretation |
| --- | --- | --- | --- |
| Uncrushed Supernatant | 1 mL water | Undetected | Below detection limit |
| Crushed Pellet | Extraction after bead-beating / 136.8 ± 14.5 ng/µL (in 200 µL) | 19.8 ± 0.4* | Robust bacterial DNA recovery |

**Note:** Data represent the mean ± SD (n = 4) derived from 20 mg of FD fecal powder.

*Each qPCR reaction was performed using a DNA template volume equivalent to 0.2 mg of the starting material.

(A) Bacterial DNA/16S rRNA gene recovery before and after mechanical crash/lysis. Bacterial DNA was almost undetectable in the pre-disruption extracellular supernatant, whereas sufficient bacterial DNA was recovered after disruption, supporting effective bacterial cell disruption.

Supplement Fig1B, table1B

| **Preparation** | **LDH activity (mU/mL)** | **Interpretation** |
| --- | --- | --- |
| Uncrushed | 0.687 ± 0.267 | Reference condition |
| Crushed | 0.588 ± 0.201 | No statistically significant difference vs. uncrushed |

**Note:** Data represent mean ± SD (n = 10).

**Statistical comparison:** paired *t*-test, *p* = 0.102; Wilcoxon signed-rank test, *p* = 0.131.

(B) LDH activity before and after the crash/lysis procedure. LDH activity (It may be of host origin (enzymes released due to damage to or inflammation of the intestinal epithelium)) did not increase and showed a slight decrease after disruption, indicating that the procedure partially affects the enzyme and highlighting the difficulty of measuring intracellular bacterial LDH in this matrix.

Supplement Fig1C

The following vitamins were evaluated for their degradation/recovery rates:

**Thiamine** (Vitamin B1) **Riboflavin** (Vitamin B2) **Nicotinic acid** (Niacin / Vitamin B3)

**Pyridoxal** (Vitamin B6) **Pyridoxine** (Vitamin B6) **Pyridoxamine** (Vitamin B6)

**Pantothenic acid** (Vitamin B5) **Biotin** (Vitamin B7) **Folic acid** (Vitamin B9)

**Methylcobalamin** (VitaminB12) **Hydroxycobalamin** (VitaminB12) **Cyanocobalamin** (Vitamin B12) **Adenosylcobalamin** (Vitamin B12)

Spike-recovery of vitamin standards before and after the crash/lysis procedure. The target low-molecular-weight vitamins were partially affected by the crushing process; therefore, the data were corrected using the recovery rates to ensure precise quantification.
