# Supplementary material for: Separating extracellular from intracellular fecal metabolites exposes cross-feeding architecture in the gut: exploring metabolite partitioning and ecological associations
Source: Gut Microbes. 2026 Aug 24;18(1):2719062. doi: 10.1080/19490976.2026.2719062 (PMC13505459; doi:10.1080/19490976.2026.2719062)
Supplement: Supplementary file 2 [file KGMI_A_2719062_SM8612.docx]

Supplemengt Fig 4:

Correlation between Type 1 Vitamins and Gut Bacteria

This figure shows the relationship between Type 1 vitamins (adenosylcobalamin, methylcobalamin, thiamine, nicotinate, and pyridoxamine)—which have high intracellular contributions—and bacterial (species) abundance. The analysis focuses on disrupted samples (total pool) as no significant correlations (|r| > 0.3) were observed in the undisrupted (extracellular) fraction for Type 1 vitamins.

Correlation between Type 2 Vitamins and Gut Bacteria

The relationship between Type 2 vitamins (riboflavin, pantothenate, and biotin)—which are considered to be secreted or present extracellularly—and bacterial species is shown.
