# Supplementary material for: Separating extracellular from intracellular fecal metabolites exposes cross-feeding architecture in the gut: exploring metabolite partitioning and ecological associations
Source: Gut Microbes. 2026 Aug 24;18(1):2719062. doi: 10.1080/19490976.2026.2719062 (PMC13505459; doi:10.1080/19490976.2026.2719062)
Supplement: Supplementary file 3 [file KGMI_A_2719062_SM8613.docx]

Supplement Fig 2: **Relationship between Clinical Findings and Gut Microbiota**

This figure illustrates the univariate correlations between clinical parameters and the abundance of gut microbiota in the study cohort (n = 63). Bacterial genera exhibiting an absolute Spearman correlation coefficient (|r|) of 0.3 or higher and a statistical significance of P < 0.05 are presented. Cells representing correlations that do not meet these criteria (absolute value below 0.3 or non-significant) are left blank. Positive correlations are indicated in red, while negative correlations are shown in blue. For the gender variable, Female is coded as 1 and Male as 0.

Left box plots show the distribution of Bifidobacterium relative abundance (%) in male (m, blue, n = 31) and female (f, red, n = 32) subjects. Statistical comparison was performed using the Mann–Whitney U test (two-sided). * p < 0.05.

Right box plots show the distribution of Bifidobacterium relative abundance (%) across milk consumption groups (0: no drink, 1: 1–3 days/week, 2: 4–6 days/week, 3: every day) for all subjects combined (n = 63). Milk consumption was treated as an ordered categorical variable. Statistical comparison across groups was performed using the Kruskal–Wallis test, followed by Dunn's post-hoc test with Bonferroni correction for pairwise comparisons.
