# Supplementary material for: Separating extracellular from intracellular fecal metabolites exposes cross-feeding architecture in the gut: exploring metabolite partitioning and ecological associations
Source: Gut Microbes. 2026 Aug 24;18(1):2719062. doi: 10.1080/19490976.2026.2719062 (PMC13505459; doi:10.1080/19490976.2026.2719062)
Supplement: Supplementary file 4 [file KGMI_A_2719062_SM8614.docx]

Supplement fig3

A

B

Supplement Fig 3: pH-Adjusted Correlations between Vitamins and Metabolites

This figure illustrates the correlations between Type 1 vitamins and SCFAs, BCFAs, and polyamines after adjusting for fecal pH

•**A:** Undisrupted samples.

**B:** Disrupted samples. The adjustment for pH more clearly separates the associations with BCFAs (upper cluster) and SCFAs (lower cluster).
